# The endogenous calpain inhibitor calpastatin attenuates axon degeneration in murine Guillain‐Barré syndrome

**DOI:** 10.1111/jns.12520

**Published:** 2022-11-18

**Authors:** Rhona McGonigal, Madeleine E. Cunningham, Duncan Smyth, Michael Chou, Jennifer A. Barrie, Andrew Wilkie, Clare Campbell, Kathryn E. Saatman, Michael Lunn, Hugh J. Willison

**Affiliations:** ^1^ School of Infection & Immunity University of Glasgow Glasgow United Kingdom; ^2^ National Hospital for Neurology and Neurosurgery, Centre for Neuromuscular Diseases University College London London United Kingdom; ^3^ Department of Physiology, Spinal Cord and Brain Injury Research Center University of Kentucky Lexington KY USA

**Keywords:** axon degeneration, calpain inhibition, Guillain‐Barre syndrome, therapeutics

## Abstract

Axon degeneration accounts for the poor clinical outcome in Guillain‐Barré syndrome (GBS), yet no treatments target this key pathogenic stage. Animal models demonstrate anti‐ganglioside antibodies (AGAb) induce axolemmal complement pore formation through which calcium flux activates the intra‐axonal calcium‐dependent proteases, calpains. We previously showed protection of axonal components using soluble calpain inhibitors in ex vivo GBS mouse models, and herein, we assess the potential of axonally‐restricted calpain inhibition as a neuroprotective therapy operating in vivo. Using transgenic mice that over‐express the endogenous human calpain inhibitor calpastatin (*hCAST*) neuronally, we assessed distal motor nerve integrity in our established GBS models. We induced immune‐mediated injury with monoclonal AGAb plus a source of human complement. The calpain substrates neurofilament and AnkyrinG, nerve structural proteins, were assessed by immunolabelling and in the case of neurofilament, by single‐molecule arrays (Simoa). As the distal intramuscular portion of the phrenic nerve is prominently targeted in our in vivo model, respiratory function was assessed by whole‐body plethysmography as the functional output in the acute and extended models. *hCAST* expression protects distal nerve structural integrity both ex and in vivo, as shown by attenuation of neurofilament breakdown by immunolabelling and Simoa. In an extended in vivo model, while mice still initially undergo respiratory distress owing to acute conduction failure, the recovery phase was accelerated by *hCAST* expression. Axonal calpain inhibition can protect the axonal integrity of the nerve in an in vivo GBS paradigm and hasten recovery. These studies reinforce the strong justification for developing further animal and human clinical studies using exogenous calpain inhibitors.

## 1 INTRODUCTION

Guillain‐Barré syndrome (GBS) is an autoimmune inflammatory peripheral neuropathy associated with varying degrees of paralysis and recovery.^1^ A poor long‐term prognosis, principally manifested by incomplete motor recovery, is due to irreversible axon degeneration, for which there is currently no treatment. In the axonal variant of GBS, acute motor axonal neuropathy (AMAN), the motor axon is directly injured leading to acute conduction failure. Clinical outcome in AMAN segregates into two extremes: reversible conduction failure (RCF) with rapid recovery or primary axon degeneration with poor recovery, although in many individual cases this dichotomisation may be mixed across nerve territories and along individual axons. RCF is understood to be a consequence of axonal conduction block at the nodes of Ranvier (NoR) and distal motor nerves in the absence of axon transection and degeneration.^2^, ^3^ In contrast, axon degeneration may be extensively distributed along the axon and, when proximally sited in the nerve roots, is associated with regeneration failure, permanent denervation and disability.^4^ The factors that dictate axonal stability and survival are currently unknown. In this regard, the concept of the “metastable state”^5^ usefully describes the tipping point beyond which an injured axon is incapable of local repair and recovery and thus undergoes the process of axon transection with ensuing Wallerian degeneration of the distal fragment. Shifting the metastable state in favour of local repair provides a useful hypothetical framework for considering axon‐protective therapeutic interventions that could improve outcomes.

Mechanisms underlying the pathogenesis of AMAN have been revealed through human autopsy studies and in animal models that attempt to recapitulate human disease pathology.^6^, ^7^, ^8^, ^9^, ^10^ Clearly, many complex factors need to be considered when interpreting both human and animal pathology.^11^ With this caveat, the prevailing view of AMAN pathogenesis is a disease mediated by complement fixing anti‐ganglioside antibodies (AGAb) induced by a preceding infection (generated by molecular mimicry in the case of *Campylobacter jejuni* infections) that bind to the peripheral nerves where gangliosides are highly enriched.^1^ Human tissue, and rabbit and mouse models of AMAN have shown complement deposition over the motor nerve terminals (MNT) and both proximal and distal motor axonal NoR. Activation of the complement cascade culminates in the formation of the membrane attack complex (MAC) pore, leading to bi‐directional movement of water and ions, including Ca^2+^ ions,^12^ which coincides with pre‐synaptic and nodal dysfunction and axonal conduction block.^8^, ^9^, ^13^, ^14^ From these models, it has been shown that at the MNT and NoR, neurofilament immunostaining, an indicator of disruption to axonal integrity preceding axon degeneration, is lost following AGAb complement‐mediated injury. Nodal and paranodal disturbances also include mislocalisation of voltage‐gated sodium (Nav) channels, cytoskeletal anchoring proteins and cell‐adhesion molecules, indicating catastrophic disruption to the nodal architecture. These effects have been successfully attenuated in animal models through classical pathway complement inhibitors^8^, ^10^, ^15^, ^16^ and multiple clinical trials in humans are underway.^17^, ^18^, ^19^

The calcium‐dependent proteases calpain I (μ‐calpain) and II (m‐calpain) are expressed in axons and selectively cleave proteins in response to physiological (micromolar, μ) or pathogenic (millimolar, m) calcium ion levels, respectively. Calpain I has a role in limited and specific proteolysis for regulatory processes, while calpain II has a role in digestive or pathologic processes. Calpain II has been implicated in neurodegeneration and axon disintegration in a variety of diseases and injury models; through the use of calpain inhibitors or calpain‐modulated transgenic mice, a role for calpains in Wallerian degeneration, Taxol‐induced sensory neuropathy and traumatic brain injury (TBI) has been revealed.^20^, ^21^, ^22^, ^23^ As we have shown a loss of known calpain substrates neurofilament, AnkyrinG and Nav channels^24^, ^25^, ^26^ in our AMAN model, we proposed that calpain activation is one major mechanism underlying axon degeneration in AMAN. We previously showed in our ex vivo mouse model, through the exogenous application of soluble calpain inhibitors, that the observed structural disturbances are mediated by activation of calpain due to the influx of Ca^2+^ ions through MAC pores.^8^, ^12^, ^27^

To explore the benefit of calpain inhibition in vivo, we assessed axonal protection using a transgenic calpastatin over‐expression paradigm. Calpastatin is a calcium‐dependent endogenous inhibitor specific for calpains^28^ which are widely expressed in the mammalian nervous system. Transgenic mice that over‐express the gene encoding human calpastatin (*hCAST*), show high expression in CNS and PNS tissue homogenates compared to wild type (WT) littermates, and by immunohistochemistry display strong calpastatin expression in neurons and axons.^20^, ^21^ Therefore, we used these mice to assess axonal structural and functional recovery in acute and extended sub‐acute AMAN mouse models.

## 2 MATERIALS AND METHODS

### 2.1 Materials

Mouse monoclonal IgG3 anti‐GD1b ganglioside antibody MOG1 and anti‐GM1 ganglioside antibody DG2, (AGAbs), were used for all experiments, as previously described and justified.^29^, ^30^, ^31^ Briefly, AGAbs were generated by immunising ganglioside‐deficient mice with ganglioside liposomes or ganglioside‐mimicking *Campylobacter jejuni* lipo‐oligosaccharide. Both AGAb bind strongly to neuronal tissue in mice, including distal motor nerves, without any notable binding to glial cells as described previously.^16^, ^32^ Normal human serum (NHS) was added as a source of complement to induce injury through AGAb‐mediated complement fixation. NHS was collected from a single donor, rapidly frozen and stored in multiple aliquots at −70°C to preserve complement activity.

The following antibodies were used for immunostaining studies to identify proteins and complement: mouse anti‐AnkG (Thermo Fisher Scientific Cat# 33‐8800, RRID:AB_2533145; 1/100); FITC‐labelled rabbit anti‐C3c (Agilent Cat# Q036805, RRID:AB_11180931; 1/300); mouse anti‐human C5b‐9 (Agilent Cat# M0777, RRID:AB_2067162; 1/50); rat anti‐MBP (Bio‐Rad Cat# MCA409S, RRID:AB_325004; 1/500); rabbit anti‐Nav1.6 (Sigma‐Aldrich Cat# S0438, RRID:AB_477480; 1/100); mouse anti‐phosphorylated neurofilament‐H antibody (NF‐H, BioLegend #801602 clone SMI31; RRID:AB_2715851; 1/1500). Secondary antibodies as follows: isotype‐specific Alexa Fluor 488‐ and Alexa Fluor 647‐conjugated goat anti‐mouse IgG3 (Thermo Fisher Scientific Cat# A‐21151, RRID:AB_2535784; 1/500), IgG2a (Thermo Fisher Scientific Cat# A‐21131, RRID:AB_2535771; 1/500) and IgG1 antibodies (Thermo Fisher Scientific Cat# A‐21240, RRID:AB_2535809; 1/500); Alexa Fluor 488‐ and Alexa Fluor 555‐conjugated goat anti‐rat IgG antibodies (Thermo Fisher Scientific Cat# A‐21434, RRID:AB_2535855; 1/500). To identify the postsynaptic membrane, fluorescently labelled alpha‐bungarotoxin (BTx) was used (Molecular Probes Cat# T1175, RRID:AB_2313931; 1/500).

For ex vivo injury preparations (described below), tissue was maintained alive in oxygenated (95% O_2_/5% CO_2_) Ringer's solution (116 mM NaCl, 4.5 mM KCl,1 mM MgCl_2_, 2 mM CaCl2, 1 mM NaH_2_PO_4_, 23 mM NaHCO_3_, 11 mM glucose, pH 7.4). Primary antibodies were applied in a blocking solution (3% normal goat serum, NGS, + 0.5% Triton‐X 100 in PBS). For diaphragm sections from in vivo experiments, antibodies were prepared in PBS plus 3% NGS + 0.1% Triton‐X 100. Secondary antibodies were diluted in PBS plus 1% NGS.

### 2.2 Mice

Three strains of mice were used: *hCAST*, *GalNAc‐T* ^*−/−*^ *‐Tg(neuronal)* (henceforth referred to as *Neuronal*) and *hCAST × Neuronal*. *hCAST* and *Neuronal* transgenic mice have previously been described. Briefly, *hCAST* mice express human calpastatin (hCAST) cDNA driven by the mouse prion protein (Prp) promoter, resulting in axonal expression throughout the nervous system.^21^ Calpastatin is a calcium‐dependent endogenous inhibitor specific for calpains.^28^ *Neuronal* mice express the full‐length cDNA encoding GalNAc‐T under the control of the human Thy1.2 promoter, restricting complex ganglioside expression (including GM1) to mature neurons.^16^ All mice were bred on a C57BL/6J background (Harlan, UK) and backcrossed for 7 generations. *hCAST* negative littermates were used as WT controls. *hCAST* were maintained as heterozygotes by breeding WT females with male *hCAST* heterozygotes. Male and female mice, ranging from 10 to 20 g, were used at 4 to 6 weeks of age, and no notable differences between genders were observed. The number of mice per treatment is reported per experiment. Mice were maintained under a 12 h light/dark cycle in controlled temperature and humidity with ad libitum access to food and water. For each study, mice were killed by rising CO_2_ inhalation. All procedures were conducted in accordance with a licence approved and granted by the United Kingdom Home Office (POC6B3485).

### 2.3 Ex vivo preparations

#### 2.3.1 Injury

Ex vivo triangularis sterni (TS) nerve‐muscle preparations were used as described previously.^14^ Briefly, TS from each mouse provide two preparations treated as follows: “Control” (AGAb only), “Injury” (AGAb plus NHS). TS were incubated for 4 h at 32°C in Ringer's with 100 μg/mL AGAb (MOG1), with the addition of 40% NHS for the injured preparation. TS were washed 3x in Ringer's followed by fixation with 4% PFA at 4°C for 20 min. Washes with PBS, 0.1 M glycine and PBS followed. TS were transferred to 100% EtOH for 10 min at −20°C then thoroughly washed in PBS. Tissue was incubated overnight at 4°C in blocking solution plus primary antibodies. TS were rinsed 3x in PBS, followed by 2 h in secondary antibodies at RT in the dark. TS were mounted in Citifluor mounting medium (Citifluor Products, UK) following final PBS washes. For each neural marker, n = 3 mice per genotype were used.

#### 2.3.2 Membrane dynamics

Temperature‐dependent internalisation studies were performed as previously described,^33^ using *hCAST* (n = 3) and WT (n = 3) mice. Briefly, TS were dissected into two halves (0 min and 60 min groups) from each animal in Ringer's solution and then incubated with 100 μg/mL AGAb (MOG1) and BTx‐555 for 2 h at 4°C. All TS were washed 3x with Ringer's. One half was immediately fixed (0 min) and the other half moved to 37°C for 1 h (60 min) to promote AGAb internalisation, followed by further washes. TS were washed and fixed as above. Tissue was incubated overnight at 4°C in PBS + 1% NGS plus secondary antibody. TS were mounted in Citifluor mounting medium following final PBS washes. For “Surface AGAb” measurements, imaging for IgG3 intensity at the MNT was performed as described below. TS were then re‐washed and incubated with 0.5% Triton X‐100 + 5% NGS for 30 min at RT to permeabilise the membrane. PBS washes, a further incubation in the same secondary Ab for 2 h at RT, washes and remounting followed. Imaging for “Total AGAb” IgG3 (surface + internalised) intensity to measure internalisation was performed.

### 2.4 Acute in vivo injury paradigm

The in vivo model used here is based on the axonal injury model previously described^10^, ^14^ with a few modifications. Briefly, WT and *hCAST* mice were habituated to whole‐body plethysmography (WBP, Electro‐Medical Measurement systems, Hampshire, UK) chambers, and the following day recordings were collected using eDacq software (version 1.9.4) to measure tidal volume (TV) as a readout of diaphragm function (Baseline). Mice received 50 mg/kg AGAb (MOG1) by intraperitoneal (i.p.) injection, followed by 30 μL/g NHS i.p. 16 h later. Naïve control littermates received no injections. WBP recordings were collected from 4‐6 h post‐NHS. Tidal volume was calculated from the average of 25 readouts for each of 25 accepted breaths at the end‐time‐point. Mice were culled at 6 h to study acute changes to distal axons. Terminal blood samples were collected and serum used for Simoa NF‐light (Quanterix Corp.) assays. Diaphragms were collected and snap frozen for immunohistological analysis. Diaphragm sections (10 and 15 μm) were collected onto APES coated slides and used for complement and calpain substrate protein analysis, respectively, at the MNT and NoR. Three slides for each marker were blocked for 1 h at 4°C, prior to antibody incubation in the same blocking solution overnight at 4°C. Slides were washed in PBS and then incubated with secondary antibodies for 2 h in the dark at RT. Slides were washed in PBS and mounted in Citifluor. For each treatment, n = 4 mice/genotype were used.

### 2.5 Sub‐acute in vivo injury paradigm

The sub‐acute injury model, described previously,^32^ was used to assess recovery. Here, we delivered AGAb targeting GM1 (DG2) to *Neuronal* mice to represent a model of AMAN.^14^ In addition, this combination of AGAb and genotype tends to have a less severe outcome and allowed the experimental window to be extended to assess recovery. Therefore, *Neuronal* and *hCAST × Neuronal* mice were injured with anti‐GM1 ganglioside antibody. This model was performed as described above with the following modifications. Mice received 40 to 50 mg/kg (Ab batch dependent) AGAb (DG2) and were survived for 96 h with careful monitoring. WBP recordings were made at 6 h, 24 h, 48 h, 72 h and 96 h. n = 4 mice per treatment. Mice were excluded from the study if their TV did not fall below 75% baseline.

### 2.6 Serum Simoa

The Simoa NF‐light assay is a digital immunoassay used to quantify NF‐L in serum, which signifies axonal damage.^34^ Simoa was performed as described previously using the Simoa Nf‐light kit in a Simoa HD‐1 Analyser (Quanterix, Lexington, MA, USA).^35^

### 2.7 Imaging and quantification

All imaging was performed using a Zeiss LSM 880 confocal microscope or Zeiss Z1 Imager with ApoTome attachment and captured with Zen software (ZEN Digital Imaging for Light Microscopy, RRID:SCR_013672). A 40x or 63x oil objective was used to capture single slices or z‐stacks (0.4 μm interval), and maximal intensity projections (MIPs) were produced for illustrative images. From these images, 17‐44 MNT, 16‐29 NoR (AnkG) and 19‐29 NoR (Nav1.6) were analysed ex vivo per treatment/mouse. 34‐95 MNT, 15‐29 NoR (AnkG) and 13‐30 NoR (Nav1.6) were analysed in vivo per treatment/mouse. Distal MNT were identified using BTx, and distal NoR were identified by a gap in MBP immunostaining. MNT and NoR occupancy was classified as occupied/present if complement or neurofilament/AnkG/Nav1.6 staining overlaid BTx or appeared between MBP domains, or absent/unoccupied. IgG3 or complement intensity overlying MNT was measured from single slices captured using consistent settings for all tissues in the same experiment.

### 2.8 Experimental design

Mice were randomly allocated to treatment groups using a random number generator. A power analysis was performed using G*Power software (3.0.10 G*Power, RRID:SCR_013726) to determine group size; n = 3 (ex vivo) and n = 4 (in vivo) were selected for each treatment group. The effect size for behavioural output was based on previous experiments, with calculations made on a basis of 80% power, and a significance criteria of 0.05. All tissue was coded prior to imaging and analysis to prevent researcher bias.

### 2.9 Statistical analysis

The numbers of independent animals are described in the Materials and Methods and indicated in the figure legends. Statistical differences were determined using GraphPad Prism 6 software (GraphPad Prism, RRID:SCR_002798). Unpaired, one‐tailed t‐tests were performed when comparing treatment without comparing genotype. Two‐way ANOVA were used for ex vivo immunoanalysis and was followed by Tukey's post‐hoc tests for multiple comparisons to compare either genotype or treatment effect. One way‐ANOVA followed by Tukey's post‐hoc test for multiple comparisons was used for in vivo immunoanalysis and Simoa data. Repeated measures two‐way ANOVA were used to analyse respiratory data followed by Sidak's or Tukey's tests for multiple comparisons to compare either genotype or time effect. Parametric testing was used, and differences were considered significant at *P* < .05. Data was plotted as the mean ± S.E.M using dotplots.

## 3 RESULTS

### 3.1 Endogenous hCAST expression protects axonal and nodal integrity in an acute ex vivo injury model

**FIGURE 1:**
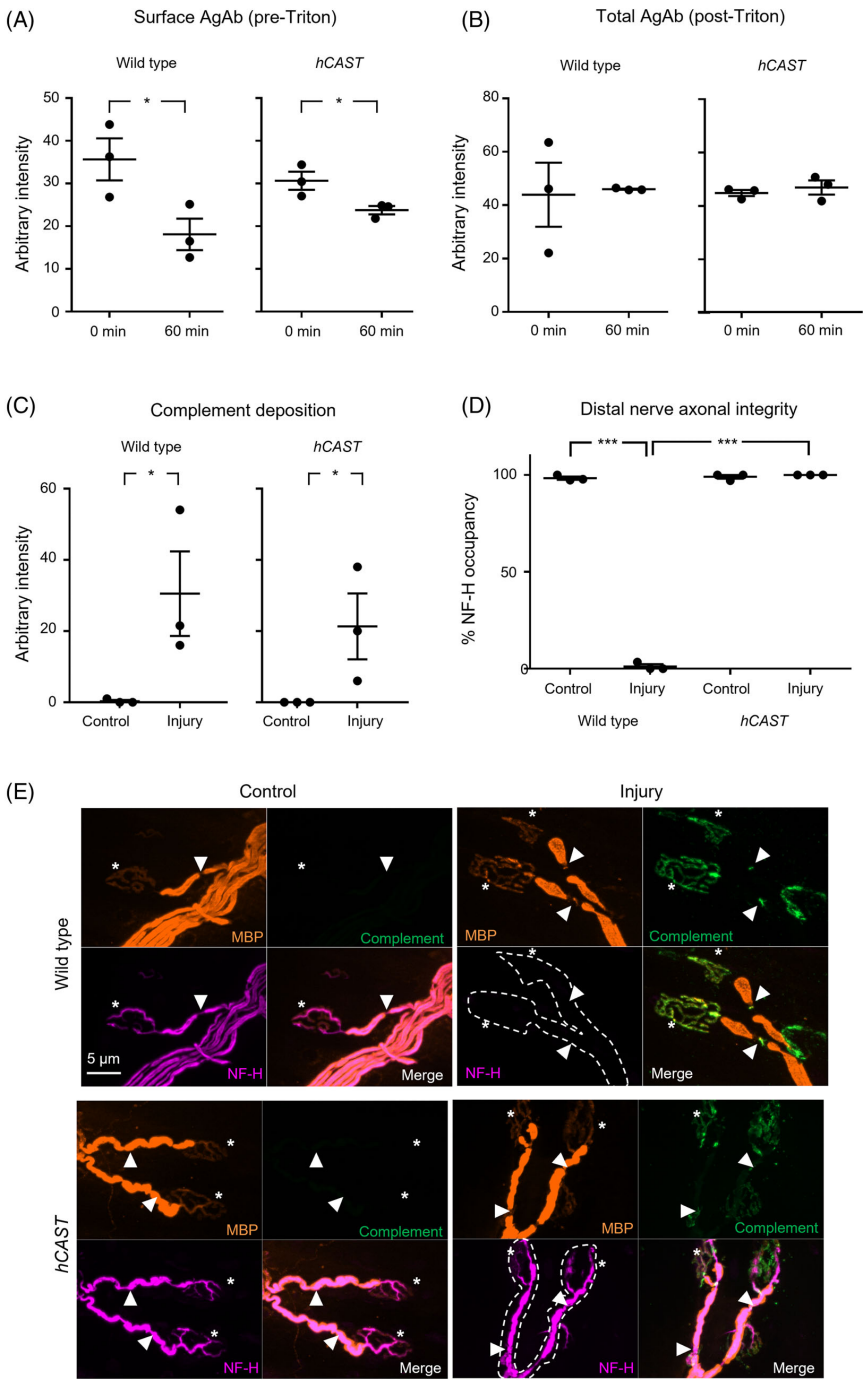
Endogenous *hCAST* expression protects axonal integrity in an acute ex vivo injury model. Triangularis sterni (TS) nerve‐muscle preparations from WT (WT, n = 3) and *hCAST* (n = 3) mice were treated ex vivo with anti‐ganglioside Ab (AGAb) for internalisation studies (A,B), and with (Injury) or without (Control) normal human serum (NHS) as a source of complement for injury studies (C‐E). A) WT and *hCAST* TS motor nerve terminals (MNT) had significantly reduced surface AGAb when incubated for 60 min at 37 °C compared to 0 min. (B) Following permeabilization with Triton X‐100, Total AGAb intensity at MNT in the 60 min group returned to normal in both genotypes suggesting internalisation of AGAb. (C) Complement (green, E) intensity at MNT significantly increased in injured WT and *hCAST* TS compared to control. (D) Neurofilament (NF‐H, magenta, E) immunostaining intensity at the MNT was significantly reduced in Injured WT tissue compared to control but was protected to control levels in *hCAST* injured tissue. (E) Illustrative images of distal nerve staining. BTx (asterisks, orange) and myelin basic protein (MBP, arrowheads, orange) were used to identify MNT and distal nodes of Ranvier (NoR), respectively. Dashed outlines indicate where NF‐H staining is absent. Scale bar = 5 μm. Dotplots = average ± S.E.M. Unpaired one‐tailed *t*‐tests were performed on data comparing treatment effect (A, B & C); * signifies *P* < .05. Two‐way ANOVA comparing treatment (control vs injury) or genotype (WT vs *hCAST*) followed by Tukey's post‐hoc multiple comparison tests were performed on data from D.; *P < .05, *** signifies *P* < .001

First, given that calpains can have a role in membrane remodelling, we assessed the membrane dynamics of *hCAST* compared to WT mouse tissue. As we have previously shown AGAb internalisation occurs at MNT when incubated at 37°C for 60 min compared to 0 min,^33^ we investigated membrane internalisation properties at this site. Here, WT MNT showed a significant reduction in surface AGAb intensity at 60 min compared to 0 min as expected (Figure 1A, unpaired one‐tailed *t*‐test, *P* < .05). *hCAST* MNT surface AGAb intensity level also showed a significant reduction at 60 min compared to 0 min (Figure 1A, unpaired one‐tailed t‐test, *P* < .05). For both WT and *hCAST*, total staining intensity was recovered to 0 min surface levels by Triton X‐100 permeabilization, which allows secondary antibody access and visualisation of intracellular AGAb (Figure 1B). These results confirm normal internalisation and suggest no detrimental effects of endogenous calpain inhibition on AGAb binding.

In our acute ex vivo model of AMAN, complement deposition at the distal nerve MNT and NoR significantly increased in injured tissue from both WT and *hCAST* mice compared to genotype control (Figure 1C,E, unpaired one‐tailed *t*‐tests, *P* < .05). In line with the AGAb binding results, this suggests *hCAST* expression does not interfere with complement activation or deposition, and therefore the initiation of injury in our model is not impaired. Next we studied MNT and NoR occupied with NF‐H immunostaining under injured conditions compared to control (Figure 1D,E, two‐way ANOVA for treatment (control vs. injury), F(1,8) = 3072 *P* < .001; for genotype (WT vs *hCAST*), F(1,8) = 3333, *P* < .001; interaction F(1,8) = 3201). In WT injured tissue, there is a significant loss of NF‐H occupancy at distal nerves, and in contrast there is no decrease in occupancy in *hCAST* injured tissue. Significantly, a *hCAST* injured nerve is protected compared to WT injured tissue.

**FIGURE 2:**
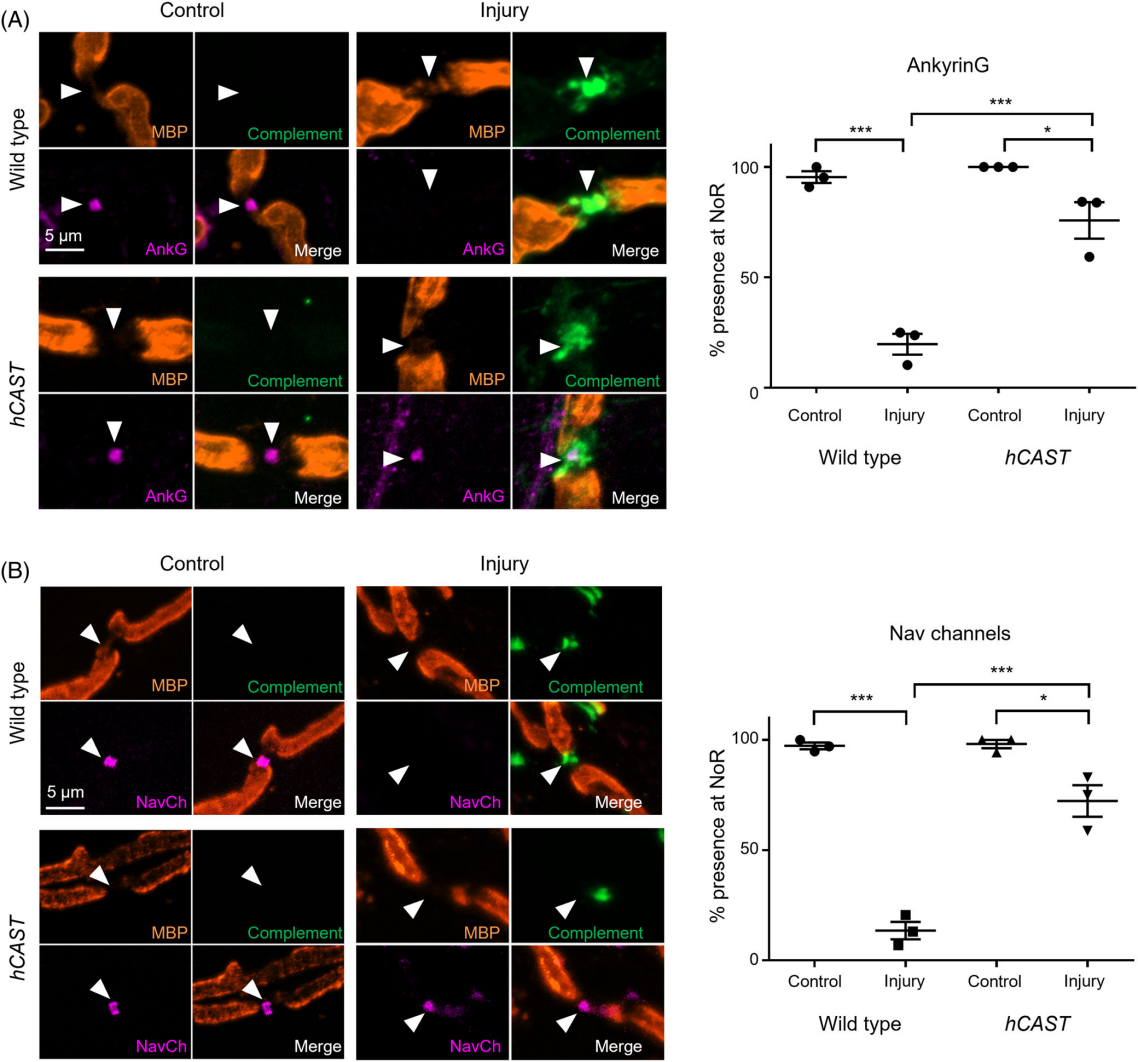
Endogenous *hCAST* expression protects nodal integrity in an acute ex vivo injury model. Triangularis sterni (TS) nerve‐muscle preparations from WT (WT, n = 3) and *hCAST* (n = 3) mice were treated ex vivo with anti‐ganglioside Ab (AGAb) with (Injured) or without (Control) normal human serum (NHS) as a source of complement. Representative images show the site of expected nodal protein immunostaining (magenta) indicated by arrowheads in the gap between myelin basic protein (MBP, orange) immunostaining. A) Significantly fewer AnkyrinG (AnkG) positive NoR were observed in WT injured tissue compared to control. This was improved by *hCAST* expression, but not to control levels. (B) NoR with Nav channel (Nav1.6) immunostaining was significantly reduced in WT injured TS compared to control; however, significant restoration occurred with *hCAST* expression. Scale bar = 5 μm. Dotplots = average ± S.E.M. Two‐way ANOVA comparing treatment (control vs. injury) or genotype (WT vs *hCAST*) followed by Tukey's post‐hoc multiple comparison tests were performed; *signifies *P* < .05, ** signifies *P* < .01, *** signifies *P* < .001

To assess the protection of structural integrity at the NoR, we next assessed AnkyrinG (Figure 2A, two‐way ANOVA for treatment (control vs injury) F = (1,8) = 102.6, *P* < .001; for genotype (WT vs *hCAST*) F(1,8) = 37.9, *P* < .001; interaction F(1,8) = 27.2, *P* < .001). In WT injured tissue, there was a significant reduction in NoR positive for AnkyrinG immunostaining compared to control. This was attenuated by *hCAST* expression: AnkyrinG positive NoR was significantly increased in *hCAST* injured tissue compared to WT injured tissue. A small but significant reduction in AnkyrinG positive NoR remained in *hCAST* injured tissue compared to *hCAST* control, showing protection was improved but incomplete. We next assessed the impact on Nav channels, which are tethered by AnkyrinG to the nodal cytoskeleton (Figure 2B, two‐way ANOVA for treatment (control vs. injury) F = (1,8) = 166.1, *P* < .001; genotype (WT vs. *hCAST*) F(1,8) = 49.1, *P* < .001; interaction F(1,8) = 46.4). Nav channel immunostaining was reduced from NoR in WT injured tissue compared to control but was protected by *hCAST* expression. Again, in *hCAST* injured tissue, a significant reduction in immunostaining remained compared to *hCAST* control, showing there was an incomplete protection.

### 3.2 Endogenous hCAST expression protects axonal and nodal integrity in an acute in vivo injury model, yet respiratory function remains impaired

**FIGURE 3:**
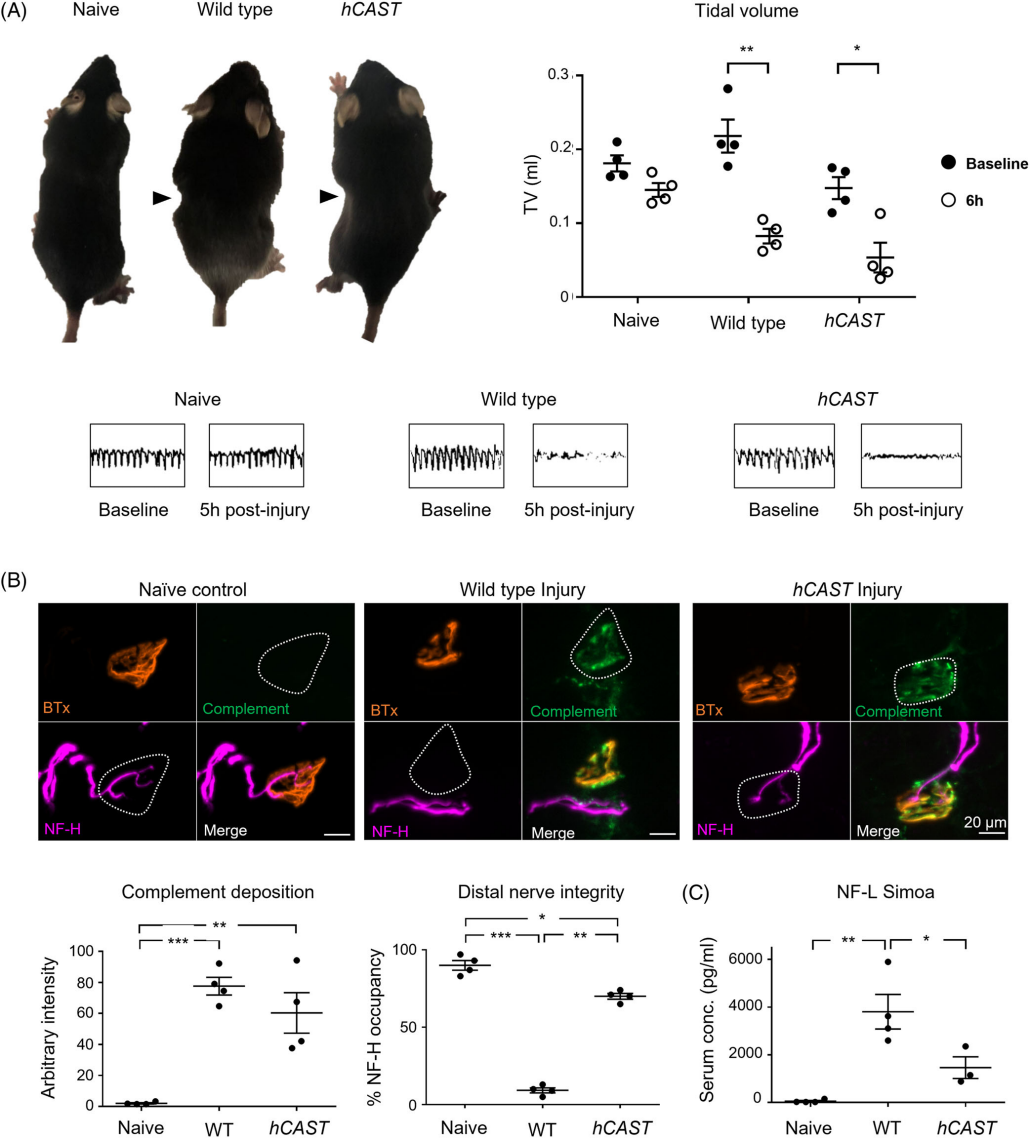
Endogenous *hCAST* expression protects axonal integrity but not function in an acute in vivo injury model. WT and *hCAST* mice were dosed i.p. with 50 mg/kg anti‐GD1b antibody (MOG1) followed 16 h later with 30 μL/g normal human serum (NHS). Naïve control littermates received no treatment. Respiratory function was monitored and diaphragm distal nerves assessed by immunoanalysis 6 h post NHS delivery. (A) Compared to baseline (black circles), at 6 h post‐injury (white circle), WT and *hCAST* mice displayed a wasp‐like abdomen (arrowheads) and a significantly reduced tidal volume (TV), measured using whole‐body plethysmography (EMMS, UK). Naïve mice showed no change. Representative respiratory flow charts for each treatment group show reduced TV in WT and *hCAST* mice. (B) Complement deposition and axonal integrity (neurofilament, NF‐H, occupancy) were compared at the diaphragm motor nerve terminals (MNT). Representative images illustrate complement deposits (green) overlying the MNT, identified by bungarotoxin (BTx, orange), in injured mice. (C) Simoa, used to measure levels of serum neurofilament light (NF‐L), showed an increase in WT injury compared to both naïve control and *hCAST* injured mice. Scale bar = 10 μm. Results are represented as the mean ± SEM, n = 4/genotype/treatment. Repeated measures two‐way ANOVA comparing group (Naive vs WT injury vs *hCAST* injury) with time (baseline vs. 6 h) followed by Sidak's post‐hoc multiple comparison tests were performed on respiratory function data. One‐way ANOVA followed by Tukey's post‐hoc multiple comparison tests were used for immuno‐ and serum analysis data. * signifies *P* < .05, ** signifies *P* < .01, *** signifies *P* < .001

In our in vivo acute mouse model of AMAN, we previously reported that following initiation of injury through intraperitoneal injection of AGAbs and complement, the diaphragm is severely compromised due to distal conduction failure, accompanied by respiratory dysfunction.^14^, ^16^ This is presumed to be due to the loss of regulation of electrophysiological function resultant from MAC pores allowing uncontrolled trans‐axolemmal salt and water fluxes, rather than axon degeneration per se. Here we report that WT and *hCAST* mice dosed with AGAb and a source of complement, NHS, display a similar wasp‐like abdomen phenotype, and severe respiratory dysfunction. Tidal volume significantly drops 6 h post‐NHS delivery compared to baseline in both WT and *hCAST mice* following injury, while there is no reduction in naïve control mice (Figure 3A, two‐way repeated measures ANOVA for group (Naïve vs. WT vs. *hCAST*) F (2,6) = 20.8, *P* < .01; time (baseline vs. 6 h) F = (1,3) = 80.4, *P* < .01; interaction F = (2,6) = 5.9, *P* < .05).

In parallel with these results, diaphragm tissue showed that complement intensity overlying the MNT significantly increased in both genotypes post‐injury compared to naïve controls (Figure 3B, one‐way ANOVA, F[2,9] = 23.09, *P* < .001). As expected, NF‐H occupancy at MNT was significantly reduced in WT injured mice in conjunction with this complement deposition (Figure 3B, one‐way ANOVA, F[2,9] = 27.3, *P* < .001). As seen in our ex vivo experiments, NF‐H occupancy was significantly improved by *hCAST* expression, with a significant increase in occupancy compared to WT injury, but occupancy did not reach naïve levels, showing incomplete protection.

NF‐L levels assessed by Simoa using serum from experimental mice supported the NF‐H immunostaining results (Figure 3C, one‐way ANOVA F[2,8] = 15.09, *P* < .01). NF‐L levels significantly increased in WT serum following injury compared to naïve controls. The levels of NF‐L in *hCAST* injured serum were significantly lower compared to WT injury and not significantly different from naïve controls. The data support our previous conclusion that functional loss is primarily electrophysiological rather than structural.

**FIGURE 4:**
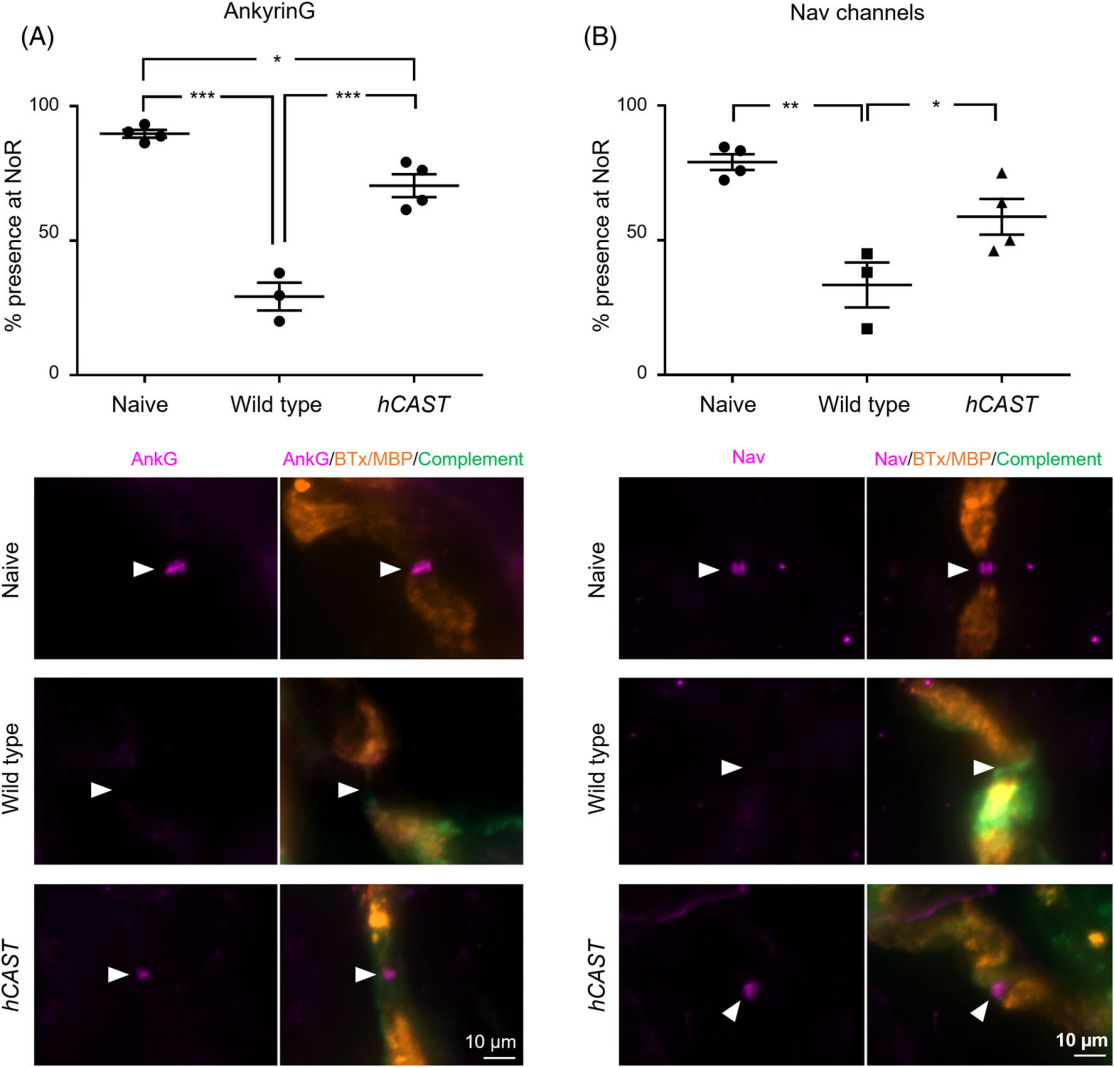
Endogenous *hCAST* expression protects nodal integrity in an acute in vivo injury model. WT and *hCAST* mice were dosed i.p. with 50 mg/kg anti‐GD1b antibody followed 16 h later with 30 μL/g normal human serum. Naïve control littermates received no treatment. Representative images show the site of expected nodal protein immunostaining (magenta) indicated by arrowheads in the gap between myelin basic protein (MBP, orange) immunostaining at the node of Ranvier (NoR). A) Significantly fewer AnkyrinG (AnkG) positive NoR were observed in WT or *hCAST* injured tissue compared to Naïve controls. *hCAST* expression significantly improved frequency compared to WT injury. (B) NoR with Nav channel (Nav1.6) immunostaining was significantly reduced in WT injured tissue compared to Naïve controls and significant protection occurred with *hCAST* expression. Scale bar = 5 μm. Dotplots = average ± S.E.M., n = 4/genotype/treatment. One‐way ANOVA followed by Tukey's post‐hoc multiple comparison tests; * signifies *P* < .05, ** signifies *P* < .01, *** signifies *P* < .001

AnkyrinG (Figure 4A) presence at the distal NoR was significantly reduced in the WT and *hCAST* diaphragm compared to naïve controls (one‐way ANOVA F[2,8] = 63.3, *P* < .001). However, NoR with AnkyrinG immunostaining did increase significantly in the *hCAST* injured diaphragm compared to the WT injured diaphragm, demonstrating the protection of this protein. A similar pattern was observed for Nav channel immunostaining (Figure 4B,F [2,8] = 13.6, *P* < .01).

### 3.3 Calpain inhibition promotes recovery in a sub‐acute model of injury

**FIGURE 5:**
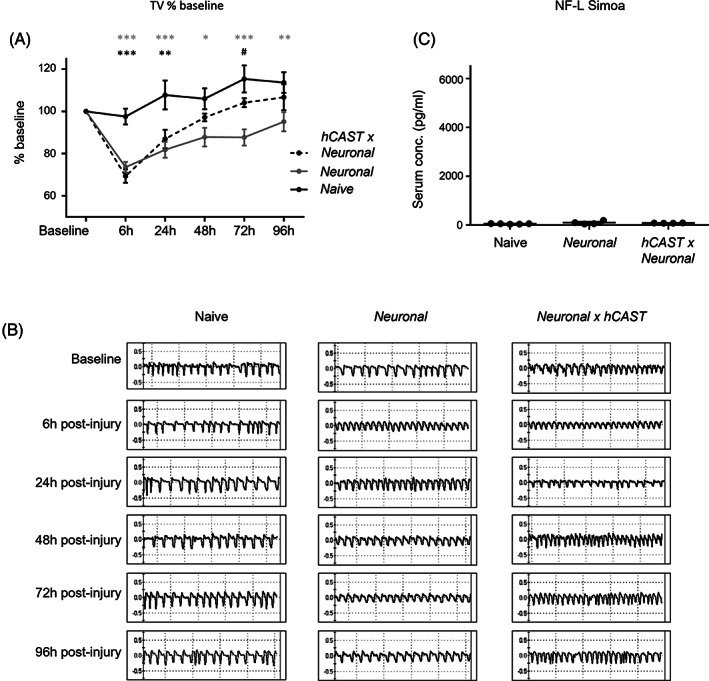
Endogenous *hCAST* expression promotes functional recovery in a sub‐acute in vivo injury model. *Neuronal* and *hCAST × Neuronal* mice were dosed i.p. with 40‐50 mg/kg anti‐GM1 antibody (DG2) followed 16 h later with 30 μL/g normal human serum (NHS). Naïve control littermates received no treatment. Respiratory function was monitored by whole‐body plethysmography (EMMS, UK) at 6, 24, 48, 72 and 96 h post NHS delivery. Compared to naïve control mice, at 6 and 24 h post‐injury both *Neuronal* and *hCAST × Neuronal* mice displayed a significant reduction in tidal volume (TV). By 48 h TV reached naïve levels in *hCAST × Neuronal* mice but remained impaired up to 96 h in *Neuronal* mice. (B) Representative respiratory flow charts for each treatment group and time‐point are shown. (C) Simoa, used to measure levels of serum neurofilament light (NF‐L), showed no differences among groups at 96 h post‐injury. Dotplot = average ± S.E.M.; n = 4/genotype/treatment. Repeated measures two‐way ANOVA comparing group (Naive vs *Neuronal* vs *hCAST × Neuronal*) with time followed by Tukey's post‐hoc multiple comparison tests were performed on respiratory function data. One‐way ANOVA followed by Tukey's post‐hoc multiple comparison tests was performed on Simoa data. * signifies *P* < .05, ** signifies *P* < .01, *** signifies *P* < .001. Black asterisks signify comparison between *hCAST × Neuronal* and naïve mice, grey asterisks signify comparison between *Neuronal* and naïve mice, and # signifies comparison between *Neuronal* and *hCAST × Neuronal* mice

As reported above, structural protection of diaphragm distal nerves is not associated with improvement in respiratory function in the acute injury model. To assess whether initial structural improvement could facilitate a faster recovery of function, we monitored mice in a sub‐acute in vivo model extending to 96 h post‐injury. Results show that there are significant changes over time that differ by genotype, and the interaction suggests a more rapid recovery by *hCAST × Neuronal* mice (Figure 5, two‐way repeated measures ANOVA for group (naïve vs. *Neuronal* vs. *hCAST × Neuronal*) F(2, 10) = 13.2, *P* < .01; time F(5, 50) = 18.3, *P* < .001; interaction F(10,50) = 3.4, *P* < .01). Initially there is a reduction in tidal volume in both *Neuronal* and *hCAST × Neuronal* mice post‐injury compared to naïve controls at 6 and 24 h. However, from 48 h, *hCAST × Neuronal* mice recover to both baseline and naïve levels, while *Neuronal* respiratory function remains impaired until 96 h. Simoa data shows that at 96 h the NF‐L serum levels following injury in both genotypes are comparable to naïve (Figure 5C, one‐way ANOVA F[2,10] = 1.84, *P* = .2) demonstrating no further neurofilament loss and a resolution of axon injury.

## 4 DISCUSSION

Axon degeneration underlies the prolonged disability and ultimately poor outcome in GBS,^11^ but there are currently no targeted treatments to counteract this feature. Using a genetic calpain inhibition strategy that yields calpastatin expression in peripheral and central neurons and axons, herein we have shown that axonal calpain leads to axon degeneration and NoR disruption in an in vivo mouse model of AMAN. Further, attenuating calpain activity can acutely protect axonal structural components and promote earlier functional recovery in an extended injury paradigm.

Aberrant calpain activation is associated with a range of neurodegenerative disorders, including but not limited to cerebral ischemia, Alzheimer's disease, Parkinson's disease and muscular dystrophies. As such, calpain inhibition as a therapeutic strategy is an established concept. Many animal models have trialled exogenous calpain inhibitors as therapeutics, and, more recently, early clinical trials have commenced,^36^ with some promising results. These studies have helped reveal the benefits and pitfalls of calpain inhibition for potentially treating GBS.

Calpain II knockout mice and exogenous application of calpain inhibitors have been successfully trialled to attenuate nervous system injury in mouse models of TBI and taxol‐induced sensory neuropathy.^22^, ^23^, ^37^ We have previously reported the success of exogenous application of soluble calpain inhibitors in attenuating structural and nodal protein cleavage in ex vivo models of AMAN.^8^, ^27^ Here, we have extended these studies to assess the efficacy of calpain inhibition in vivo. The development of calpain inhibitors has been hampered by limited water solubility, cell permeability, metabolic stability and specificity.^36^ Currently, the endogenous calpain inhibitor calpastatin is the only selective inhibitor for calpains, although strategies to improve the specificity of exogenous inhibitors are being developed.^36^ Therefore, we elected to use transgenic mice that over‐express the gene for human calpastatin (*hCAST*) under control of the Prp promoter, resulting in widespread neuronal *hCAST* expression in both peripheral and central nervous systems, inclusive of axons, at levels as much as 80‐fold higher than endogenous mouse calpastatin.^21^ It has been reported that *hCAST* mice display a normal phenotype, and by immunolabelling with a calpastatin antibody, Ma et al.^20^ showed calpastatin levels are increased in transgenic peripheral nerves and MNT compared to WT. In addition, it was reported that *hCAST* mouse sciatic nerves were structurally protected in a WD transection paradigm. The *hCAST* mouse benefits from site‐specific calpain inhibition with no off‐target toxicity from calpain inhibition at other sites and eliminates the need to deliver calpain inhibitors globally or implant invasive delivery pumps into our mice. Here we showed that we could recapitulate our ex vivo results using *hCAST* mice, and additionally that axonal neurofilament and nodal proteins AnkyrinG and Nav channels were protected in our acute in vivo AMAN model.

### 4.1 Incomplete structural protection and mis‐matched functional improvements

In this transgenic model, the dose of calpastatin is fixed and therefore cannot be controlled or altered in the way that soluble inhibitors could be. Calpastatin reversibly binds and blocks calpain, and levels are likely not enough to completely protect all calpain‐mediated cleavage.^28^ Indeed, we see a partial protection of immunostaining for the Nav channel and structural proteins NF‐H and AnkyrinG in both our ex and in vivo models. NFH and AnkG are known calpain substrates^38^ [Boivin, 1990 #222], and Nav channels are either cleaved directly^25^ or mis‐localised through cleavage of their AnkyrinG tether. The additional disturbances in protein localisation we observed could also be attributed to other cytotoxic mediators or physiological changes such as inflammatory oedema or membrane swelling caused by the presence of MAC pores and associated water influx. Calpain is involved in membrane remodelling under normal physiological conditions^28^; therefore, it is possible that complement pore shedding from the axolemmal membrane could be impaired in the presence of calpain inhibition. Nevertheless, we did not find any changes to AGAb binding or internalisation, or indeed complement deposition, in our paradigms. In subjects with GBS, calpain therapeutics would be delivered after disease onset; even if membrane dynamics were altered in an unexpected way by calpain inhibition, the short‐term effects would likely be minimal, and the benefits would more likely outweigh any subtle impairments.^36^

Intriguingly, despite the structural protection observed in our acute model, nerve function remained impaired. MAC pore complement deposition remained present; therefore, we interpreted as a failure of membrane potential homeostasis since free movement of water and ions will occur bi‐directionally through MAC pores and impair resting membrane potential and thereby function.^8^ Here, we describe similar results in our acute in vivo model. In the initial stages of injury, physiological function is not protected, but early structural protection appears to ultimately promote a more rapid functional recovery. It is possible that this reduces the likelihood of axon degeneration and poor outcomes through a shift in the metastable state in favour of protection.^5^ Critically, it is important to appreciate that inexcitable axons do not directly equate to degenerating axons; therefore, early axon‐protective intervention could attenuate axon degeneration and improve axon fate, currently a major gap in knowledge.

Protection of structure but failure to prevent conduction loss was also observed after sciatic nerve transection in *hCAST* mice^20^ and in optic nerves exposed to anoxia in the presence of pharmacologic calpain inhibitors.^39^ Despite this lack of functional recovery following transection, normal endogenous calpastatin expression levels in WT mice are unable to prevent injury caused by calpain activity in an acute nerve transection model. Whereas the authors report successful reduction in NF‐L and NF‐H proteolysis and cytoskeletal preservation in the sciatic nerve from over‐expressing *hCAST* mice up to 5 days. It was proposed for the latter models that other calcium‐dependent processes could potentially mediate this persistent loss of function. In the same transgenic mice, Schoch et al.^21^ found an attenuation of calpain‐mediated cleavage of structural protein α‐spectrin, voltage gated sodium channels, and collapsin response mediator protein‐2 in brain tissues after TBI. In a neuron‐specific calpastatin overexpressing mouse, similar attenuation of calpain‐mediated proteolysis coincided with behavioural improvements in a model of TBI.^40^ Intriguingly, TBI models using exogenous calpain inhibitors demonstrate functional improvements, but with more limited success in lessening proteolysis or neuron death.^41^, ^42^, ^43^ Variable responses in calpain inhibitor drug studies may be related to complexities in delivery in the correct time‐window, dosing frequency, or titration of dosages.^44^

Therefore, the benefit of complement and calpain inhibitor delivery after the onset of injury is critical and remains to be assessed in GBS models. However, we propose that the “tipping point” of the axon in the metastable state hypothesis^5^ could be shifted in favour of repair (rather than transection) by later delivery. Indeed, we report here the accelerated functional recovery of mice where axons are protected early. Additionally, a combinatorial therapeutic approach using both complement and calpain inhibition would likely enhance patient outcome and is an avenue worth exploring in future studies. Use of these transgenic mice is a simplified system, and thus the trial of exogenous calpain inhibitors in human GBS would be the crucial next step.

### 4.2 Serum protein levels to predict therapeutic efficacy

It has been shown from patient samples that long‐term prognosis in GBS patients can be predicted by serum levels of NF‐L, with higher cleavage indicating a poor outcome.^35^, ^45^ Interestingly, serum biomarkers can also be associated with neuropathy‐related inflammatory oedema and are a more sensitive marker than electrophysiological abnormalities.^11^ Quantitative clinical assessment is a major unmet need in understanding treatment efficacy, and the use of a response biomarker would be beneficial for future trials.^34^ For the first time in an animal model, corresponding with immunostaining loss, we show the axon breakdown product NF‐L in the serum as a useful readout of axon injury and clinical disease. After a single acute insult, NF‐L levels increased, and 96 h later, normalised to naïve levels, suggesting no ongoing injury. Most significantly, we used this method to show attenuation of axon damage with calpain inhibition. This occurred despite acute functional loss and coincided with a faster functional recovery in an extended injury paradigm. Due to blood volume collection restrictions in experimental mice, we were not able to measure serum NF‐L at every time‐point. However, we have previously reported using immunostaining that NF‐H and MAC, along with function as assessed by WBP, return to normal 72 h after sub‐acute injury in WT mice *in vivo*, suggesting some relationship between axon integrity and function exists.^32^ Therefore, based on these initial findings, it is possible that serum levels could correspond with function and be used for clinical assessment. These results suggest serum NF‐L levels could be used to assess the efficacy of drug treatment in future models and substantiate the use of serum assays to predict prognosis in patients. Indeed, biomarkers of axonal injury are likely to be more useful than clinical assessment, as electrophysiological function, and thus clinical performance, may be highly dissociated from structural integrity in the acute phase of illness, as discussed above.

### 4.3 Conclusions and future directions

This *in vivo* study of calpain inhibition in an AMAN model provides a proof of principle for calpain modulatory therapies in future animal and human studies. Early axon protection will likely promote recovery in all patients, according to the metastable state hypothesis outlined above. Recent unpublished evidence from our animal studies also points towards calpain involvement in secondary axon degeneration (unpublished observations); therefore, calpain inhibition and consequent axon protection could be universally beneficial to all variants of GBS in which primary and secondary axonal degeneration occur and has encouraging implications for therapeutic strategies aimed at enhancing long‐term recovery.

## AUTHOR CONTRIBUTIONS

Rhona McGonigal and HJW devised the study and wrote the manuscript. Rhona McGonigal, Madeleine E. Cunningham, Duncan Smyth, Michael Chou, Jennifer A. Barrie performed experiments. Andrew Wilkie and Clare Campbell provided experimental support. Kathryn E. Saatman and Michael Lunn provided critical feedback. All authors revised the final manuscript.

## CONFLICT OF INTEREST

The authors have declared that no conflict of interest exists.

## Data Availability

The data supporting the findings reported in this paper are available from the University of Glasgow Enlighten:Research Data repository at https://doi.org/10.5525/gla.researchdata.1360.
